# NKT cells are important mediators of hepatic ischemia-reperfusion injury

**DOI:** 10.1016/j.trim.2017.08.002

**Published:** 2017-12

**Authors:** James A. Richards, Stephen J. Wigmore, Stephen M. Anderton, Sarah E.M. Howie

**Affiliations:** a MRC Centre for Inflammation Research, The University of Edinburgh, Edinburgh, UK; b Clinical Surgery, The University of Edinburgh, Edinburgh, UK

**Keywords:** DAMPs, Danger Associated Molecular Patterns, DCD, donation after cardiac death, IFN, interferon, IgM, immunoglobulin M, IL, interleukin, IRI, ischemia-reperfusion injury, MHC, major histocompatibility complex, NK, natural killer, NKT, natural killer T cell, SEM, standard error of the mean, TCR, T cell receptor, Th, T helper cell, TReg, regulatory T Cells (CD3 + CD4 + FoxP3 +), TNF, tumour necrosis factor, Acute liver injury, Ischemia-reperfusion injury (IRI), T cells, NKT cells, Natural killer cells, Transplantation, Liver surgery

## Abstract

**Introduction:**

IRI results from the interruption then reinstatement of an organ's blood supply, and this poses a significant problem in liver transplantation and resectional surgery.

In this paper, we explore the role T cells play in the pathogenesis of this injury.

**Materials & methods:**

We used an in vivo murine model of warm partial hepatic IRI, genetically-modified mice, in vivo antibody depletion, adoptive cell transfer and flow cytometry to determine which lymphocyte subsets contribute to pathology. Injury was assessed by measuring serum alanine aminotransfersase (ALT) and by histological examination of liver tissue sections.

**Results:**

The absence of T cells (CD3εKO) is associated with significant protection from injury (*p* = 0.010). Through a strategy of antibody depletion it appears that NKT cells (*p* = 0.0025), rather than conventional T (CD4 + or CD8 +) (*p* = 0.11) cells that are the key mediators of injury.

**Discussion:**

Our results indicate that tissue-resident NKT cells, but not other lymphocyte populations are responsible for the injury in hepatic IRI. Targeting the activation of NKT cells and/or their effector apparatus would be a novel approach in protecting the liver during transplantation and resection surgery; this may allow us to expand our current criteria for surgery.

## Highlights

• Hepatic IRI worsens outcome in liver transplantation.
• T cells are important in hepatic IRI.
• These are tissue-resident rather than recruited T cells.
• NKT, but not conventional T or NK cells, are key mediators of hepatic IRI.
• Targeting NKT activation or their effector apparatus may offer therapeutic potential.

## 1 Introduction

Liver failure is increasing dramatically in the UK population [1]. Currently, liver transplantation is the only effective treatment for patients with end-stage disease, giving an average of 17–22 years of additional life [1], [2], [3]. Ischemia-reperfusion injury (IRI) limits access to liver transplantation and is linked to early graft failure [4], [5], [6]. Marginal organs including those from older or steatotic donors are particularly susceptible to IRI [7]. The problem of marginal organs is becoming more prevalent and results from changes in donor profile and a rise of donation after cardiac death (DCD) [8]. IRI also poses a significant challenge in the contexts of major liver resection surgery [9] and hypoxic hepatitis often seen in critically ill patients [10].

IRI results from the interruption then reinstatement of an organ's blood supply. The initial ischemic injury leads to disruption of cellular integrity with the release of Danger Associated Molecular Patterns (DAMPs). DAMPs initiate a secondary (immune-mediated) response within the liver. This immune response causes further collateral damage to cells that may have otherwise survived the primary ischemic injury [11], [12]. Mice deficient in both B and T cells are protected from hepatic IRI [13], [14], [15]. We have previously demonstrated that it is T cells, not B cells (or IgM), that are the key mediators of injury [15]. This fits with work from other laboratories, identifying a variety of “key” T cells populations [16], [17], [18]. It should be noted that Caldwell et al. found CD4 + lymphocytes to be protective in their model of IRI [19]; at least in our hands, we could not attribute this protection to CD4 + Foxp3 + regulatory T cells (TReg) [20].

There is a significant enrichment of non-conventional T cells within the liver [21], [22], [23], with Natural Killer T (NKT) cells accounting for up to 30% of the resident intrahepatic lymphocytes. Hepatic NKT play an important role in hepatic immunosurveillance [24] and are linked with both immunosuppressive and pro-inflammatory responses [25]. They are capable of rapidly (within 1–2 h) producing large amounts of pro-inflammatory cytokines, including interferon-gamma (IFNγ), IL-4 and TNFα [26], [27].

In this paper, we explore the role T cell subsets play in the pathogenesis of hepatic IRI.

## 2 Materials and methods

### 2.1 Ethical approval & animal welfare

Following local ethical approval at the University of Edinburgh, animal work was carried out according to UK Home Office regulations (Animals Scientific Procedures Act 1986) under licenses 60/4045. Mice were housed under specific pathogen-free conditions at the University of Edinburgh. All wild type (WT), knockout and transgenic mice were age-matched male mice on a C57BL/6J background. RAG1 −/− mice lack both mature B and T cells [28]. CD3εKO mice lack mature T cells [29]. > 95% of CD4 + T-cells in OT-II mice express αβ-T Cell Receptors (TCR) recognising chicken ovalbumin [30]. IL-17RA −/− mice lack the IL-17 Receptor alpha chain and are unresponsive to both IL-17 (A-F) and IL-25 [31]. General anaesthesia (GA) was induced via inhaled isoflurane and post-operatively subcutaneous opioid analgesia (buprenorphine) was administered. Animals were sacrificed under GA by terminal exsanguination by way of cardiac puncture.

### 2.2 Surgical model of hepatic IRI

In this model of warm partial hepatic IRI, an atraumatic clamp was applied to the vascular pedicle supplying the left lobe for 20–50 min; the liver was then allowed to reperfuse for up to 24 h. Intraoperative core body temperature was maintained at 36 °C with a homeothermic blanket system (Harvard Apparatus, Edenbridge, UK) to minimise the masking effects of hypothermia on liver IRI [32].

The extent of any liver injury was assessed in terms of the serum level of alanine aminotransfersase (ALT), a biochemical marker of liver injury [33]. ALT was measured on a Cobas Fara centrifugal analyser (Roche Diagnostics Ltd., Welwyn Garden City, UK) using a commercially available kit (Alpha Laboratories Ltd., Eastleigh, UK). ALT was correlated with the histological evidence of injury seen on sections of formalin fixed tissue stained with haematoxylin and eosin (H&E).

### 2.3 In vivo antibody depletion

#### 2.3.1 CD4 and CD8 depletion

We have previously used monoclonal antibodies YTS169.4.2.1 and YTS191.1.1.2 in vivo to deplete CD8 + and CD4 + cells respectively [34]. Mice were given an intraperitoneal (i.p.) injection of 50μg CD8 depleting antibody (YTS 169.4.2.1) or 100μg of CD4 depleting antibody (YTS 191.1.1.2) or isotype control (AFRC MAC 51) made up to 150 μl in PBS at days -6 and days -2 prior to surgery; antibodies were sourced commercially (Immunosolv, Edinburgh, UK). This regimen gave 99% depletion of CD8 + T cells in the spleen and liver using YTS 169.4.2.1 and 91% of CD4 + T cells using YTS 191.1.1.2 (data not shown); this is consistent with that established in the literature [34], [35].

#### 2.3.2 NK and NKT depletion

PK136 (BioXcell, West Lebanon, USA) was used to deplete NK1.1 + cells. On a C57BL/6 background this depletes both NK and NKT cells [36], [37]. Mice were given an i.p. injection of either 100μg PK136 or mouse IgG2a isotype control (C1.18.4, BioXcell) 48 h pre-operatively. This gave a depletion of 91% CD3_int_NK1.1 + cells (data not shown) in line with that described in the literature [36], [37], [38].

NK cells were selectively depleted by anti-asialo GM1 antibody [39]. Mice received i.p. injection of 30 μl reconstituted anti-asialo GM1 antibody (Wako, Neuss, Germany) or an equivalent volume of rabbit serum (Sigma-Aldrich) at day -1 relative to surgery. Similar to reports in the literature [38], [40], [41], this regimen gave a depletion of over 90% NK cells (data not shown).

### 2.4 Flow cytometry

Single cell preparations from the liver were generated by a combination of mechanical disruption (GentleMACS, Miltenyi Biotec, Bisley, UK) and enzymatic digestion (2 mg/ml Collagenase D, Roche). These were then passed through a 70 μm filter and centrifuged at 50*g* for 5 min to remove debris and hepatocytes. Red cells were lysed (Red Cell Lysis Buffer, Sigma-Aldrich, Poole, UK). Immune cells were isolated by positive selection using a CD45 + MicroBead AutoMACS separation (Miltenyi Biotec, Bisley, UK) and then stained with a fixable Live-Dead marker (Life Technologies, Paisley, UK) and a multi-colour panel of antibodies, including CD3, CD4, CD8, CD19 and NK1.1 (Biolegend, San Diego, USA). NKT cells were also identified using PBS-57 tetramers, an analogue of alpha-galactosylceramide developed by Dr. Paul Savage (The NIH Tetramer Facility, Emory University, Atlanta, USA) and complexed to CD1d tetramers [42].

Samples were then run on a BD LSR II Fortessa (BD Biosciences, Oxford, UK) and analysed with FlowJo software (Tree Star, Ashland, USA). T cells were defined as CD3 + CD19 − cells and NKT cells as CD3intNK1.1 + (or CD3 + Tetramer +) cells, within a forward-side scatter defined lymphocyte gate (Supplemental Fig. 1).

### 2.5 Adoptive transfer

Isolation of lymphocytes from spleen was performed by mechanical disaggregation through a 40 μm filter. Cells were transferred either as mixed populations (e.g. “splenic lymphocytes”), or after purification with AutoMACS CD4 + MicroBeads, using previously published cells transfer protocols [43], [44].

### 2.6 Immunohistochemistry

Tissue from the models of murine liver injury was fixed in methacarn (60% methanol, 30% chloroform, 10% glacial acetic acid (Sigma-Aldrich)). Sections were deparaffinised and rehydrated before endogenous peroxidase and avidin/biotin activity were quenched, prior to incubating with a rat monoclonal anti-mouse Ly6g antibody (Ab25377, Abcam, Cambridge, UK) at a dilution of 1 in 100. Slides were subsequently incubated at room temperature with polyclonal rabbit anti-rat biotinylated secondary antibody (E0468, DAKO, Ely, UK) at 1 in 400 dilution for 40 min. Sections were then developed with VectaStain RTU Elite (Vector Laboratories, Peterborough, UK) followed by diaminobenzidine (DAB125, Spring Biosciences, Pleasanton, UK), before being counterstained.

### 2.7 Statistical analysis

Groups were analysed with the aid of Prism 5 for Mac OSX (Graphpad Software, La Jolla, USA); specific statistical methods are referred to in the results section. All values in graphs represent mean ± standard error of the mean (SEM) unless stated otherwise.

## 3 Results

### 3.1 T cells play a central role in the secondary immune-mediated injury

**Fig. 1:**
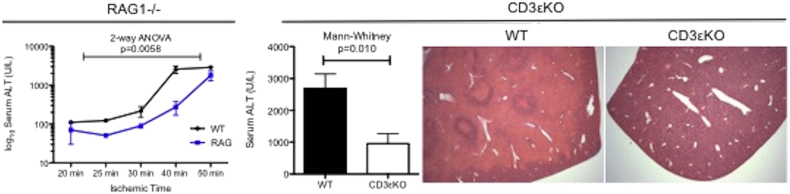
T cells are key mediators of warm hepatic IRI.

RAG1 −/− mice are deficient in both mature B and T cells and were significantly protected from experimental hepatic IRI across a range of ischemic injuries (Fig. 1). There was significant protection in RAG1 −/− mice up to a point where the observed injury in RAG1 −/− and WT converged; this corresponded to complete ischemic necrosis within this model. T cell deficient (CD3εKO) mice were also significantly protected from injury (Fig. 1).

WT and RAG1 −/− mice underwent 20–50 min of warm left lobe hepatic ischemia and were reperfused for 24 h. There was significant protection in RAG1 −/− mice (which lack IgM, T and B cells) compared to WT controls (Kruskal-Wallis *p* = 0.0058, *n* > 3 per timepoint). We had previously shown this was not as a result of B cells (or IgM) [15]. Mice lacking T cells (CD3εKO) or WT controls underwent 40 min ischemia and were then reperfused for 24 h (*n* = 12 per arm). There was significant biochemical protection (Mann-Whitney *p* = 0.010) in CD3εKO mice; this corresponded with histological protection (representative H&E stained sections × 25 magnification).

### 3.2 Tissue-resident rather than recruited T cells are responsible for injury

**Fig. 2:**
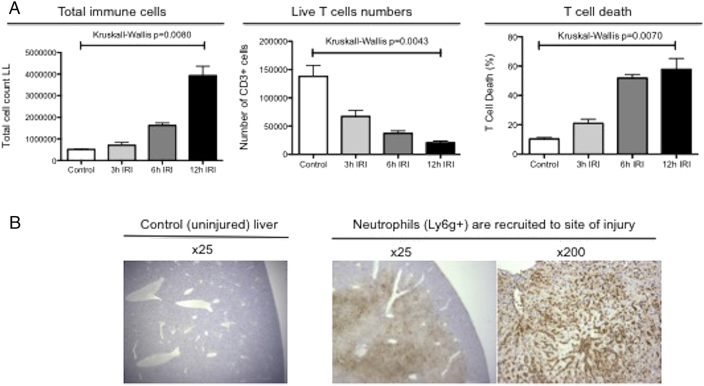
There is no significant influx of T cells into the liver during IRI.

Following ischemic injury, there was a significant and rapid influx of immune cells (defined as CD45 +) into the post-ischemic lobe (Fig. 2A); these were predominantly (Ly6g +) neutrophils (Fig. 2B). With increasing time there was a decrease in the number of viable T cells found within the post-ischemic liver and no significant mobilisation of T cells following reperfusion injury. Taken together with the protection seen in CD3εKO mice (Fig. 1), this points to tissue-resident (rather than recruited) T cells being key mediators of the secondary immune-mediated injury.

To look at whether there was a change in the immune composition following IRI, WT mice were exposed to 40 min left lobe ischemia and reperfused for 3, 6 or 12 h (*n* = 4/group). T cells were defined as CD3 + CD19- gated lymphocytes. The immune cells isolated within the ischemic lobe were initially analysed by flow cytometry. There was a significant influx of immune cells (defined as CD45 + cells) into the injured left lobe (Kruskall-Wallis *p* = 0.0080) (**A**). The number of viable T cells was found to decrease significantly with time following reperfusion (Kruskall-Wallis *p* = 0.0043); this was predominantly due to cell death (as defined by positive staining for a live-dead marker) (Kruskall-Wallis *p* = 0.0070) (**A**). Few neutrophils are found in uninjured areas of liver, but are a major component of the immune cell influx following IRI (**B**). Here neutrophils are defined by immunohistochemistry as cells positively staining for Ly6g (representative section shown).

### 3.3 Hepatic IRI is independent of IL-17

**Fig. 3:**
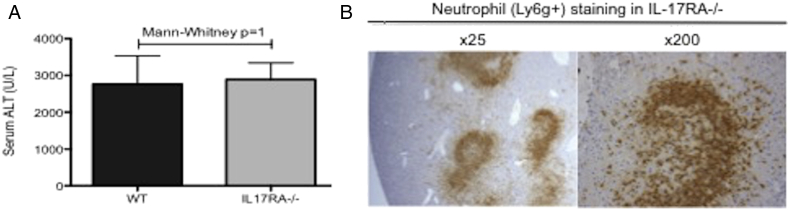
Hepatic IRI and recruitment of neutrophils is independent of IL-17 and IL-25.

Interleukin-17 (IL-17) has been linked with the pathology of a number of autoimmune diseases [45] and is important in neutrophil chemotaxis [46]. Given the significant role of T cells (Fig. 1) and recruitment of neutrophils to the site of liver injury (Fig. 2B), it was proposed that Th17 cells, which are an important source of IL-17, may play a significant role in the pathology of this injury. IL-17RA −/− mice had no reduction in injury compared to WT controls (Fig. 3A); this implied that neither IL-17 (A-F) nor IL-25 play a critical in the pathogenesis of hepatic IRI. Neutrophils were recruited to the site of injury in these mice, implying that IL-17 was not an essential chemoattractant of neutrophils in this setting (Fig. 3B).

Given role for T cells and the influx of neutrophils (Fig. 1, Fig. 2), mice deficient in IL-17 Receptor subunit A (IL-17RA −/−) (*n* = 5/group) were used to see whether there was a significant role for Th17 cells in the context of this injury. There was no significant protection (Mann-Whitney *p* = 1) seen in IL-17RA −/− mice compared to WT controls following 40 min ischemia and 24 h reperfusion (**A**). Furthermore, there was recruitment of neutrophils into sites of liver injury following IRI in IL-17RA −/− mice (**B**) (representative section shown), suggesting that neither IL-17 nor IL-25 are critical to the recruitment of neutrophils in this model.

### 3.4 CD4 + TCR specificity has no significant bearing on injury

**Fig. 4:**
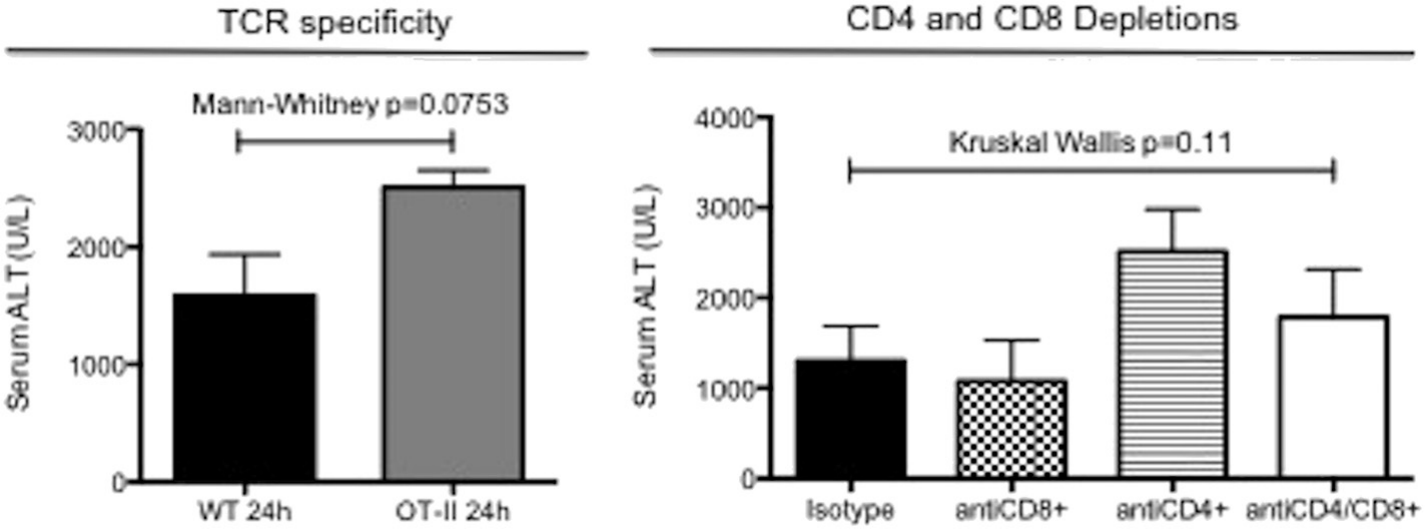
Conventional T cells are not important in hepatic IRI.

Others have previously implicated CD4 + T cell activation via an antigen-dependent process as playing a significant role in injury [37]. OT-II mice have > 95% of the TCR on CD4 + T cells specific for chicken ovalbumin [30]. It was therefore proposed that, if TCR recognition of tissue expressed antigen was important in IRI, these mice would be protected from injury. There was no protection observed in OT-II mice compared to WT controls (see Fig. 4).

OT-II mice have > 90% of the TCR on CD4 + T cells specific for chicken ovalbumin. OT-II mice and WT controls underwent 40 min ischemia and 24 h reperfusion; there was no significant difference in the observed injury (*n* = 5 per group, representative data from 2 experiments). To assess the role of conventional (CD4 + and CD8 +) T cells, mice were injected with isotype control (*n* = 10) or CD4 (*n* = 10), CD8 (*n* = 9) or CD4 & CD8 (*n* = 4) depleting antibodies at days -6 and -2 prior to surgery (40 min ischemia followed by 24 h reperfusion). Despite significant depletion of CD4 + and/or CD8 + T cells (as detailed in methods), there was no significant reduction in the biochemical evidence of injury compared to isotype control (Kruskal-Wallis *p* = 0.10).

While this implied that conventional activation of CD4 + T cells is not important, there may be a role for CD8 + T cells or non-classical CD4 + T cells activation. To examine the roles of CD4 + or CD8 + T cells in hepatic IRI, systemic antibody depletion of CD4 + and CD8 + cells was performed. Despite significant depletion of CD4 + (83%) and CD8 + (99.9%) cells there was no significant reduction in injury. To exclude there being sufficient redundancy in the system, both CD4 + and CD8 + cells were depleted synchronously (86%), and still there was no reduction in the observed injury (*p* = 0.11) (Fig. 4).

Together with the protection in CD3εKO mice (Fig. 1), this implied that neither conventional CD4 + nor CD8 + T cells are significantly involved in the pathogenesis of the observed injury. This would also fit with the observation that adoptive transfer of splenic CD4 + T cells, which are mainly conventional, failed to reconstitute injury (Supplemental Fig. 1). Hepatic NKT cells have an intermediate expression of TCRs compared to their conventional counterparts and can be either CD4 +, CD8 + or CD4/CD8 double negative [24].

### 3.5 Depletion of NKT cells reduces injury

**Fig. 5:**
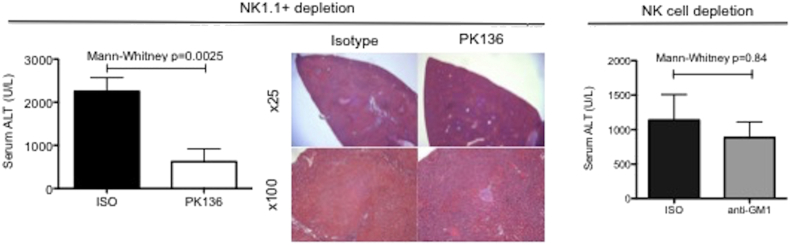
NKT (but not NK) cells are key mediators of warm hepatic IRI.

We found that NKT cells are significantly enriched within the liver, but account for only a minor population of T cells within the spleen (Supplemental Fig. 2); this fits with data in the published literature [21]. Mice were treated with anti-NK1.1 antibody, which depletes both NK and NKT cells. There was a significant reduction in the observed injury (*p* = 0.0025) (Fig. 5). Data from RAG1 −/− and CD3εKO mice (Fig. 1), both having an intact NK compartment, but lacking NKT cells, suggested that NK cells do not play a significant role in experimental IRI. To test this, NK cells were selectively depleted in WT mice with anti-asialo GM-1 antibody; there was no observed protection, despite > 90% depletion of NK cells (Fig. 5).

Mice were injected i.p. 48 h pre-operatively with 100μg PK136 or mouse IgG2a isotype control (ISO); on a C57BL/6 background this depletes both NK and NKT. The average depletion of CD3_int_ + cells by PK136 was 91% compared to the isotype control. Mice were subjected to a 40 min ischemic injury and reperfused for 24 h (*n* = 8). Mice treated with PK136 were significantly protected from hepatic IRI (Mann-Whitney *p* = 0.0025). To exclude this being as a result of NK cells, mice were injected at day-1 with 30 μl anti-asialo GM1 antibody or rabbit serum to selectively deplete them. This led to a significant depletion of 90–97% NK cells. However, following 40 min ischemia and 24 h reperfusion; there was no significant difference in the biochemical evidence of injury (representative data from one experiment (*n* = 5 per group), Mann-Whitney *p* = 0.84).

## 4 Discussion

While conflicting data exist within the literature about the protective or pathogenic nature of CD4 + cells [16], [17], [19], we looked directly at the role T cells play in hepatic IRI. The absence of T cells (CD3εKO mice) was associated with significant protection from injury (Fig. 1). Work using TCR −/− mice has been performed in models of renal IRI, where T cells were also found to be key mediators of injury [47], [48]. We have previously published that B cells [15] and regulatory T cells play no significant role in this model or warm hepatic IRI [20]. Others have reported T cells to be essential for cerebral [49], [50], cardiac [51], pulmonary [52], mesenteric [53] and renal IRI [47], [48], indicating common mechanisms in IRI irrespective of the organ involved.

The timeframe in which IRI occurs (minutes to hours) precludes a “classical” (conventional) T cell response involving clonal expansion and migration of effector populations to the site of injury (days). Work presented here using mice with a restricted CD4 + TCR repertoire (Fig. 4), combined with data from Khandoga et al., in which they found no reduction in injury following in vivo antibody blockade of MHC II [16], suggests that the observed pathogenic role of T cells in IRI cannot result from classical epitope-specific peptide: MHC-TCR mediated activation. This implicates innate rather than conventional T cells in the pathogenesis of hepatic IRI. The lack of a role for conventional CD4 + T cells in hepatic IRI together with the IL-17RA −/− data (Fig. 3), confirms that Th17 cells (along with IL-17 and IL-25) are not critical to this injury or neutrophil recruitment to the site of injury. This fits with work from others, who have shown that neutrophils are directly recruited by danger signals (e.g. adenosine triphosphate) to focal areas of liver necrosis [54].

NKT cells are significantly enriched within the liver [22], [23]. In this study, we found they accounted for 10–15% of all lymphocytes, compared to < 1% in the spleen (Supplemental Fig. 2). Hepatic lymphocytes express characteristic chemokine receptors, which play a critical role in the trafficking of T cells through the liver, maintaining tissue resident populations of T cells and in liver inflammation [55], [56], [57], [58]. Given the differences in the baseline characteristics of hepatic and splenic T cells, it is perhaps not surprising that the adoptive transfer experiments using splenic-derived lymphocytes in an acute model of liver injury failed to recapitulate injury (Supplemental Figs. 1 and 3), even before their specificity, activation status and expression of trafficking receptors are considered.

Kuboki et al. also found a significant role for NKT cells in their in vivo experiments utilising anti-CD1d blocking antibodies (to block NKT activation) and PK136 depleting antibody (to deplete all NK1.1 + cells) [37]. The reduction of injury, through the blockade of CD1d, points to this being via conventional activation of NKT cells (as opposed to via non-TCR receptors) [37]. The work we present here extends that of Kuboki et al., by specifically showing that NK cells (which are also NK1.1 + on a C57BL/6 background) do not play a significant role in hepatic IRI (Fig. 5). Further evidence for this comes from the significant protection observed in RAG1 −/− mice (Fig. 1), which have functional NK and innate lymphoid cell compartments. The timescale involved also fits with NKT cells being the important population of T cells in IRI, as they are capable of rapidly producing large amounts of pro-inflammatory cytokines [26], [27]. The speed at which NKT cells produce cytokines is thought to be related to pre-formed intracellular stores of mRNA leaving them “poised for action” [59].

Most liver transplant surgeons will decline a liver for transplantation if it is deemed at risk of significant IRI. This is because of concerns about early allograft dysfunction, which is associated with a worse short-term mortality as well as poorer 1, 3 and 5 year graft and patient survival [60], [61], [62], [63]. Liver-resident NKT cells have probably evolved to recognise liver damage arising from the release of DAMPs caused by fulminant hepatic infections. Understandably they cannot distinguish that scenario from the 21st century concepts of organ transplantation or liver resection surgery.

Machine organ perfusion (such as ex-vivo normothermic perfusion (EVNP)) is an emerging technology, which restores circulation to a retrieved organ prior to transplantation [64]. The implication of the findings presented in this paper is that by simply putting a lymphocyte filter into the machine perfusion circuit to “filter off” lymphocytes may not be sufficient to provide substantial protection from IRI. This is because it is the tissue-resident (not circulating) NKT population that are key to injury and will need to be targeted pharmacologically rather than mechanically. This technique of capturing circulating donor leucocytes during machine perfusion, may have longer term benefits in terms of modulating the allogenic immunogenicity of the transplant [65], [66]. Beyond machine perfusion our work also suggests that immunotherapy solely targeting the recruitment of T cells into the liver is unlikely to have a significant impact in combating IRI.

While further work to dissect out the exact nature of NKT cell activation in hepatic IRI is also required, it does suggest that targeting the activation of NKT cells and/or their effector apparatus either in vivo or during ex-vivo perfusion, could be a better and novel approach to minimising the (immune-mediated) secondary liver injury seen. Based on the window of protection we see in immunodeficient mice (Fig. 1), the use of cytoprotective strategies alone (such as up-regulating of heme oxygenase-1 (HO-1) [67], [68], [69]) may only give limited protection due to the effects of the secondary immune response. Furthermore we showed no matter how potent an immunosuppressive treatment strategy is, this can only be used effectively as a treatment when the index ischemic event is “survivable”.

Immunomodulatory and cytoprotective therapeutic strategies are, therefore, likely to be best used in combination and may in fact be synergistic. Effective therapies against IRI may allow us to expand our criteria for liver transplantation (as well as resection surgery) and decrease early organ dysfunction along with its long-term sequelae.

## Funding

This study was made possible by the support of The Wellcome Trust (WT 092494/Z/10/Z), The Royal College of Surgeons of Edinburgh (Maurice-Wohl MWRF/10/002 & RCSEd Small Grant), Tenovus Scotland (E09/8) and The Dowager Countess Eleanor Peel Trust.

## Disclosures

The authors who have taken part in this study declared that they do not have anything to disclose regarding funding or conflict of interest with respect to this manuscript.

## Author contributions

JR, SW, SA & SH jointly conceived and designed the study. JR obtained the funding for this study, acquired/analysed/interpreted all of the data and drafted the original manuscript. SW, SA & SH supervised the study and provided critical revision/editing of the final manuscript.

## Appendix A Supplementary data

Supplementary material Image 1
