# Supplementary material for: NKT cells are important mediators of hepatic ischemia-reperfusion injury
Source: Transpl Immunol. 2017 Dec;45:15–21. doi: 10.1016/j.trim.2017.08.002 (PMC5694034; doi:10.1016/j.trim.2017.08.002)
Supplement: Supplementary file 1 — Supplementary material [file mmc1.docx]

**Supplemental Data**

**
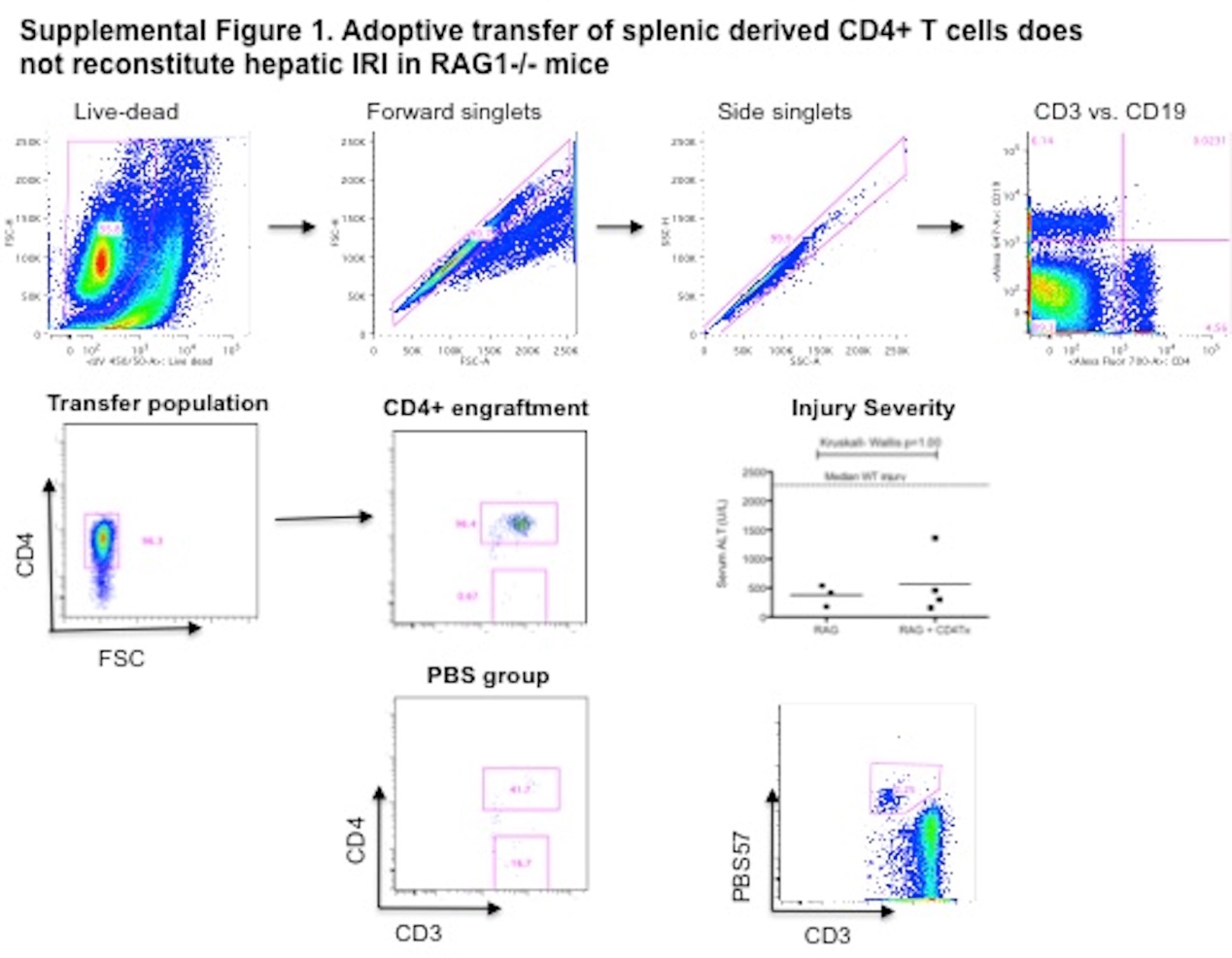
**

**Supplemental Figure 1. Adoptive transfer of splenic derived CD4+ T cells does not reconstitute hepatic IRI in RAG1-/- mice**

6x10^6^ CD4+ splenic lymphocytes with a purity of >95% were adoptively transferred into RAG1-/- recipients 24 hour pre-operatively. Mice underwent hepatic IRI (40 minutes of ischemia followed by 24 hours of reperfusion). There was no significant difference in the observed injury (Mann-Whitney p=1.00). Successful engraftment of cells within the mice was assessed within the spleen; there were so few cells in the PBS spleens that they needed to be pooled. There is a clear population of CD3+CD4+ cells in those receiving a cell transfer unlike in the PBS control group.

**
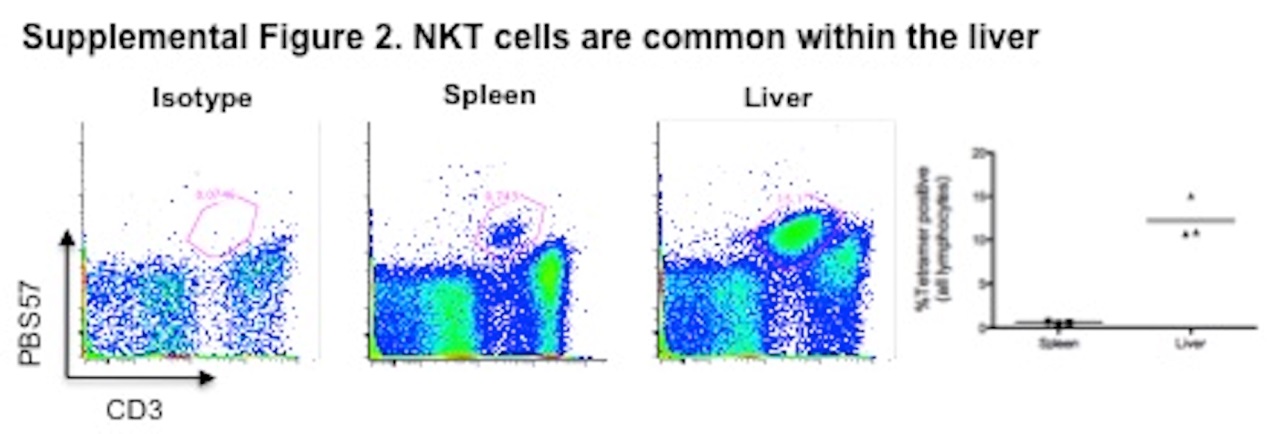
**

**Supplemental Figure 2. NKT cells are common within the liver**

The frequency of NKT cells (defined as CD3int NK1.1+ Tetramer+) within the liver was determined by flow cytometry. There was relative enrichment of NKT in the liver compared to the spleen in WT mice (representative flow plots).


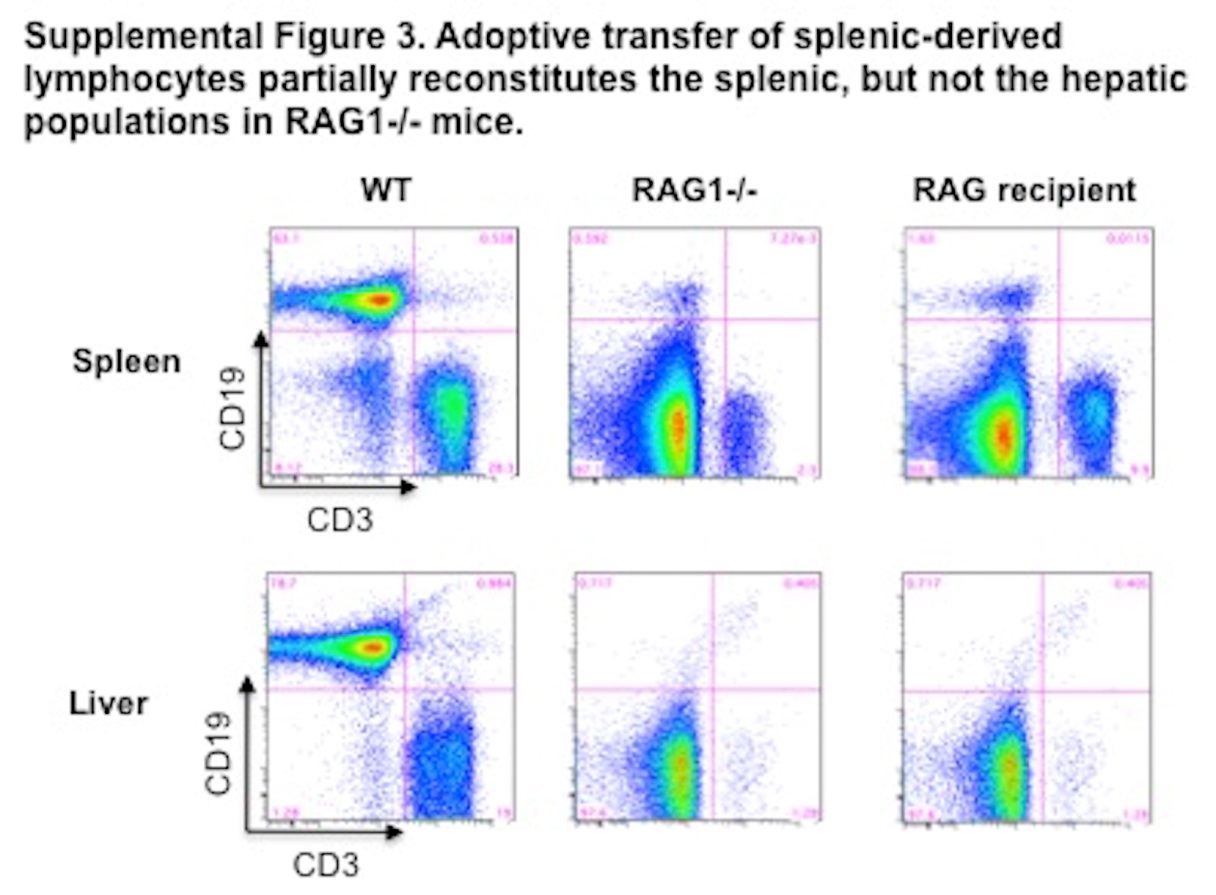


**Supplemental Figure 3. Adoptive transfer of splenic-derived lymphocytes partially reconstitutes the splenic, but not the hepatic populations in RAG1-/- mice.**

To assess whether adoptive transfer of splenic lymphocytes is a good method for repopulating RAG1-/- mice in a model of acute liver injury requiring tissue resident T cells, 3.5x10^7^ lymphocytes derived from the spleen of WT donor mice were injected i.p. into RAG1-/- recipients. The transfer population of cells was representative of a WT spleen. RAG1-/- contained very few lymphocytes, with NK cells accounting for over 90% of all lymphocytes, compared to 4-6% in WT mice (data not shown). With adoptive transfer there was partial reconstitution of the splenic population of T and B cells, but not in the liver (representative flow plots).
